# Cytosolic, but not matrix, calcium is essential for adjustment of mitochondrial pyruvate supply

**DOI:** 10.1074/jbc.RA119.011902

**Published:** 2020-02-24

**Authors:** Marten Szibor, Zemfira Gizatullina, Timur Gainutdinov, Thomas Endres, Grazyna Debska-Vielhaber, Matthias Kunz, Niki Karavasili, Kerstin Hallmann, Frank Schreiber, Alexandra Bamberger, Michael Schwarzer, Torsten Doenst, Hans-Jochen Heinze, Volkmar Lessmann, Stefan Vielhaber, Wolfram S. Kunz, Frank N. Gellerich

**Affiliations:** ‡ Faculty of Medicine and Health Technology, Tampere University, FI-33520 Tampere, Finland; § Department of Cardiothoracic Surgery, Jena University Hospital, D-07747 Jena, Germany; ¶ Department of Neurology, Otto-von-Guericke-University, D-39120 Magdeburg, Germany; ‖ Leibniz-Institute for Neurobiology, D-39120 Magdeburg, Germany; ** Research Institute for Problems of Ecology and Mineral Wealth Use, Tatarstan Academy of Sciences, Kazan 420087, Russia; ‡‡ Institute of Physiology, Otto-von-Guericke-University, D-39120 Magdeburg, Germany; §§ Institute of Experimental Epileptology and Cognition Research, University of Bonn, D-53127 Bonn, Germany; ¶¶ German Center for Neurodegenerative Diseases, D-39120 Magdeburg, Germany; ‖‖ Center for Behavioral Brain Sciences (CBBS), D-39120 Magdeburg, Germany

**Keywords:** mouse, mitochondria, respiratory chain, calcium, bioenergetics, malate-aspartate shuttle, mitochondrial calcium uniporter, cytosolic calcium, OXPHOS control, synaptosomes, thymocytes, fibroblasts, isolated working rat heart

## Abstract

Mitochondrial oxidative phosphorylation (OXPHOS) and cellular workload are tightly balanced by the key cellular regulator, calcium (Ca^2+^). Current models assume that cytosolic Ca^2+^ regulates workload and that mitochondrial Ca^2+^ uptake precedes activation of matrix dehydrogenases, thereby matching OXPHOS substrate supply to ATP demand. Surprisingly, knockout (KO) of the mitochondrial Ca^2+^ uniporter (MCU) in mice results in only minimal phenotypic changes and does not alter OXPHOS. This implies that adaptive activation of mitochondrial dehydrogenases by intramitochondrial Ca^2+^ cannot be the exclusive mechanism for OXPHOS control. We hypothesized that cytosolic Ca^2+^, but not mitochondrial matrix Ca^2+^, may adapt OXPHOS to workload by adjusting the rate of pyruvate supply from the cytosol to the mitochondria. Here, we studied the role of malate-aspartate shuttle (MAS)-dependent substrate supply in OXPHOS responses to changing Ca^2+^ concentrations in isolated brain and heart mitochondria, synaptosomes, fibroblasts, and thymocytes from WT and MCU KO mice and the isolated working rat heart. Our results indicate that extramitochondrial Ca^2+^ controls up to 85% of maximal pyruvate-driven OXPHOS rates, mediated by the activity of the complete MAS, and that intramitochondrial Ca^2+^ accounts for the remaining 15%. Of note, the complete MAS, as applied here, included besides its classical NADH oxidation reaction the generation of cytosolic pyruvate. Part of this largely neglected mechanism has previously been described as the “mitochondrial gas pedal.” Its implementation into OXPHOS control models integrates seemingly contradictory results and warrants a critical reappraisal of metabolic control mechanisms in health and disease.

## Introduction

Synchronization of cellular workload and mitochondrial energization (*i.e.* balancing cytosolic ATP-consuming processes and mitochondrial oxidative phosphorylation (OXPHOS)^3^ of ADP for ATP production) is a vital necessity, and calcium (Ca^2+^) has been identified as a key regulatory molecule (1–3). Current models assume that substrate supply for OXPHOS is controlled by mitochondrial Ca^2+^ uptake and subsequent activation of matrix enzymes such as pyruvate dehydrogenase (PDH), α-oxoglutarate dehydrogenase and isocitrate dehydrogenase (1, 4). Doubts as to the validity of this mechanism arose, among other reasons, from the application of mathematical models (5, 6) and the flux control theory (7, 8), which indicate that an increase of a single enzyme activity may, but must not necessarily, increase the total metabolic flux. Indeed, in heart mitochondria isolated from rats kept on a regular diet, it was demonstrated that the Ca^2+^ dependence of PDH is measurable only at the enzyme level and not at the level of OXPHOS fluxes (4). Doubts were further reinforced when knockout (KO) of the mitochondrial Ca^2+^ uniporter (MCU) in the mouse revealed only minimal physiologic abnormalities and regular OXPHOS in heart mitochondria despite a lack of short-term mitochondrial Ca^2+^ uptake (9–15). Also, in brain mitochondria isolated from MCU KO mice, Ca^2+^ uptake was found to be substantially diminished, albeit not entirely abolished (14, 16), and elevated cytosolic Ca^2+^ levels in smooth muscle cells and fibroblasts (17) as well as in cardiomyocytes (13) from MCU KO mice were interpreted as an indicator for a missing activity of the MCU for cellular Ca^2+^ clearance. Notwithstanding the overwhelming evidence contradicting current models of metabolic homeostasis under physiologic conditions, the regulatory role of mitochondrial matrix Ca^2+^ has not been questioned. Instead, disputable alternative Ca^2+^ uptake routes have been proposed as an underlying regulatory mechanism (9–16), but the problem of how MCU KO mice avoid an energy crisis has not yet been conclusively solved (18).

In contrast to others (1, 4, 9–15) and based primarily on work in brain mitochondria, we hypothesized that cytosolic but not mitochondrial matrix Ca^2+^ may adapt OXPHOS activity (19–29), and we gave experimental evidence that the malate-aspartate shuttle (MAS) plays an essential role in providing mitochondria with pyruvate generated in the cytosol, a model previously described as the “mitochondrial gas pedal” (19–22). Ca^2+^ sensitivity of the MAS is known to be facilitated by the mitochondrial glutamate aspartate carrier, Aralar (23–27), which is an essential MAS component with a regulatory Ca^2+^-binding site facing the mitochondrial intermembrane space. Therefore, the MAS (with respect to its Ca^2+^-sensitive component Aralar) senses cytosolic but not mitochondrial matrix Ca^2+^ levels (23–26), and thus Aralar-mediated cytosolic Ca^2+^ sensing may explain why the MCU is largely dispensable for OXPHOS control under physiologic conditions. This, however, has not yet been experimentally demonstrated, because most previous studies on isolated mitochondria (25, 28) suffer from technical shortcomings. Most importantly, pyruvate-regenerating reactions, which are inseparably connected to MAS activity, have so far been largely neglected. As a consequence, most studies to date have restricted substrate supply to hydride anions derived from NADH oxidation and thus dramatically underestimated the actual effect of the MAS. In support of our hypothesis, work in cortical neurons, in which Aralar was genetically silenced, demonstrated that activation of MAS/Aralar is a necessity for pyruvate generation and supply to activate OXPHOS (26). This study, however, did not discriminate the proportional impact of cytosolic and/or mitochondrial matrix Ca^2+^ on both workload and OXPHOS. Another confounding factor complicating the comparability of previous results is that different organs and species might depend to different degrees on MAS activation and MCU-mediated Ca^2+^ uptake.

To overcome the aforementioned limitations and to dissect the Ca^2+^-dependent signaling pathways matching workload and OXPHOS, we established the “complete MAS assay” and used Ca^2+^ chelators and specific inhibitors in experimental models of different origin and with increasing biological complexity. The models used side-by-side in this study include isolated mouse brain and heart mitochondria, synaptosomes and intact primary cells, and the working rat heart. Furthermore, to identify the compartment in which Ca^2+^ sensing controls OXPHOS, we took advantage of mice with genetic MCU ablation (MCU KO). The combination of MCU KO mice with the methodologically refined reconstituted, complete MAS assay allowed us to unequivocally unmask the OXPHOS control mechanisms that so far had escaped accurate assessment. Our data establish the complete MAS as the major provider of pyruvate for OXPHOS regulated by cytosolic, but not mitochondrial matrix, Ca^2+^ in the different models. This integrates seemingly contradictory results on Ca^2+^-mediated OXPHOS control and may provide the intellectual basis for novel therapeutic approaches to diseases where cell death is initiated by mitochondrial Ca^2+^ overload.

## Results

### Validation of the MCU KO mouse model

To study the effect of Ca^2+^ from different cellular compartments on the regulation of OXPHOS, we took advantage of an MCU KO mouse model established by Pan *et al.* (9). We confirmed previous findings that the mitochondrial Ca^2+^ uptake is practically absent in MCU KO mitochondria from heart and dramatically diminished in brain mitochondria as compared with WT littermates. These findings were controlled by the use of the MCU inhibitor ruthenium red (RR) (Fig. S1, *A–D* (brain) and *E–H* (heart)). Using RT-PCR analysis, we detected residual *Mcu* transcripts in MCU KO mice (Fig. S2, *A* and *B*), reaching 7.3% in hippocampus and 3.9% in heart tissue as compared with WT (Fig. S2, *C* and *D*). The observed residual transcripts may result from unintended gene-trap splicing (Fig. S2*B*) and may account for an observed small, but bioenergetically negligible, extent of Ca^2+^ uptake by MCU KO brain mitochondria (less than 3% as compared with WT (Fig. S1, *C* and *D*)). Although not followed up specifically, both the residual MCU activity and other MCU-independent Ca^2+^ uptake pathways may be equally responsible for the minimal matrix Ca^2+^ content seen in MCU KO mice (9, 10). To test for potential bioenergetic consequences of impaired mitochondrial Ca^2+^ uptake, we measured substrate-specific rates of mitochondrial respiration. Notably, MCU KO mitochondria from brain and heart did not reveal a significant difference (Fig. S3, *A* and *C*). A decrease in ADP-stimulated phosphorylating respiration triggered by Ca^2+^ overload, as seen in WT, was absent in MCU KO mitochondria and/or treatment with RR (Fig. S3, *B* and *D*). We also examined whether the MCU KO-related subtle bioenergetic alterations affect the mouse phenotype in the given genetic background. Visual comparison revealed no abnormalities other than a small but significant decrease in body weight in MCU KO mice (Fig. S4*A*), confirming earlier results (9). As an indicator for intact brain metabolism, we used behavioral tests and assessed orientation and learning processes (*i.e.* open field tests (Fig. S4, *B–D*) and Morris water maze tests (Fig. S4, *E–G*)). Generally, WT and MCU KO mice exhibited similar learning performances across all training trials except for a small but significantly decreased swim velocity of MCU KO mice (Fig. S4*F*). Taken together, our data confirm that MCU KO effectively impairs mitochondrial Ca^2+^ uptake while leaving the mouse physiology generally unaffected. This makes the MCU KO mouse strain an excellent model to study mitochondrial OXPHOS in response to free Ca^2+^ levels in different cellular compartments.

### Pyruvate-driven OXPHOS is only minimally activated by the addition of Ca^2+^

**Figure 1.**
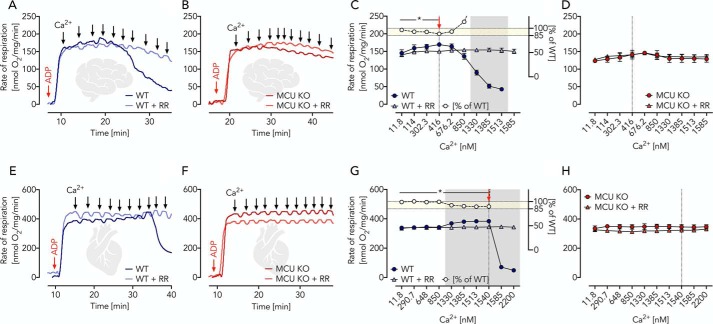
**Ca^2+^-independent pyruvate/malate-driven respiration in brain and heart mitochondria isolated from WT and MCU KO mice.** *A* and *B*, respiratory traces of WT and MCU KO brain mitochondria in EGTA media supplemented with pyruvate, malate, ADP, and RR as indicated; *arrows* specify Ca^2+^ additions for a stepwise increase from ∼12 nm to 2 μm measured fluorimetrically using Fura-2. *C*, respiratory rates of WT brain mitochondria in the presence and absence of RR as indicated shown as mean ± S.E. (*error bars*) of *n* = 5 experiments plotted against free Ca^2+^ concentrations. The *red arrow* indicates significantly increased Ca^2+^-induced respiration in WT mitochondria (but not WT + RR) determined using two-way ANOVA with Dunnett's multiple-comparison post hoc test. *, *p* < 0.05. *Gray area*, significant difference between WT and WT + RR mitochondria determined using two-way ANOVA analysis with Sidak's multiple-comparison post hoc test and a *p* value of <0.05. *White circles* visualize the percentage of WT + RR respiration compared with WT calculated from the mean values (*yellow area* denotes 85–100% of WT respiration). *D*, respiratory rates of MCU KO brain mitochondria in the presence and absence of RR, as indicated, shown as mean ± S.E. of *n* = 5 experiments plotted against free Ca^2+^ concentrations. *E* and *F*, respiratory traces of WT and MCU KO heart mitochondria in EGTA media supplemented with pyruvate, malate, ADP, and RR as indicated; *arrows* specify Ca^2+^ additions for a stepwise increase from ∼12 nm to 2 μm. *G*, respiratory rates of WT heart mitochondria in the presence and absence of RR as indicated shown as mean ± S.E. of *n* = 5 experiments plotted against free Ca^2+^ concentrations. The *red arrow* indicates significantly increased Ca^2+^-induced respiration in WT mitochondria (but not WT + RR) determined using two-way ANOVA with Dunnett's multiple-comparison post hoc test. *, *p* < 0.05. *Gray area*, significant difference between WT and WT + RR mitochondria determined using two-way ANOVA with Sidak's multiple-comparison post hoc test and a *p* value of <0.05. *White circles* visualize the percentage of WT + RR respiration compared with WT calculated from the mean values (*yellow area* denotes 85–100% of WT respiration). *H*, respiratory rates of MCU KO heart mitochondria in the presence and absence of RR as indicated shown as mean ± S.E. of *n* = 5 experiments plotted against free Ca^2+^ concentrations.

**Figure 2.**
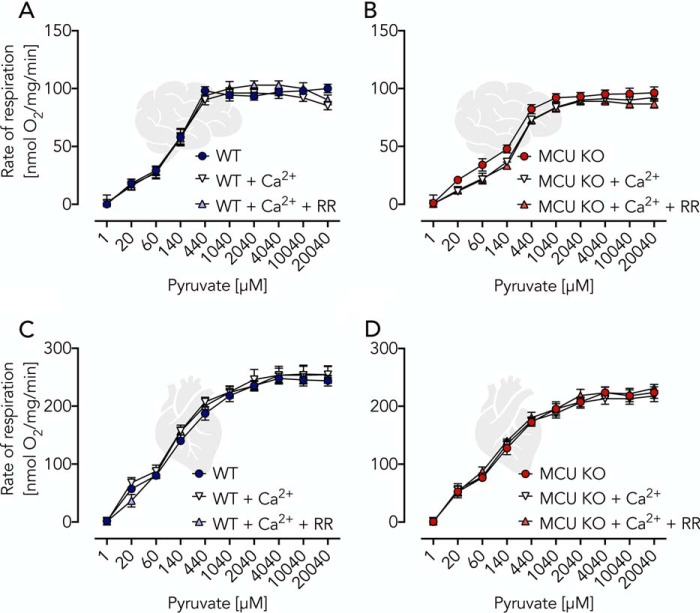
**Validation of Ca^2+^ independence of pyruvate-induced respiratory activation using isolated brain and heart mitochondria.** *A* and *B*, respiratory rates of WT and MCU KO brain mitochondria (0.06 mg/ml) incubated in EGTA buffer supplemented with ADP (2 mm) and malate (2 mm) and additions of pyruvate, Ca^2+^ (800 nm), and RR as indicated. *C* and *D*, respiratory rates of WT and MCU KO heart mitochondria (0.04 mg/ml) under conditions as described for *A* and *B*. All data are shown as mean ± S.E. (*error bars*) of *n* = 5 experiments.

Pyruvate is a chief substrate for OXPHOS in brain mitochondria mainly generated by the complete MAS (19, 20). Its oxidation is executed by PDH in a Ca^2+^-sensitive reaction (1, 4), which led to the assumption that this is the key mechanism to adapt OXPHOS activity to cellular workload. We studied OXPHOS in isolated mitochondria fed with pyruvate-malate as a substrate combination. Ca^2+^ levels were controlled using incubation buffers supplemented with a 1 mm concentration of the Ca^2+^ chelator EGTA. This strategy allowed accurate Ca^2+^ titration experiments by repeated additions of Ca^2+^. Levels of free Ca^2+^ were monitored using the fluorescent reporter Fura-2 detecting free Ca^2+^ concentrations ranging from ∼12 nm to 2 μm (Fig. 1, *A–D* (brain) and *E–H* (heart)). To adjust optimal OXPHOS conditions, saturating amounts of ADP (2 mm final concentration) were added, making the OXPHOS activity dependent on the rate of substrate oxidation (state 3). In mitochondria isolated from WT brain and heart tissue, maximal Ca^2+^-stimulated respiratory rates (*V*_max_ = 100%) were reached at Ca^2+^ concentrations of 0.4 μm (brain) and 1.5 μm (heart) (*red arrow* in Fig. 1, *C* and *G*). Of note, already prior to Ca^2+^ additions, mitochondrial respiratory rates reached ∼85% of *V*_max_ and remained insensitive to Ca^2+^ stimulation when the MCU inhibitor RR was present. A similar observation was made previously in heart mitochondria prepared from rats kept on a standard diet (4). In essence, this means that only ∼15% of *V*_max_ was sensitive to mitochondrial matrix Ca^2+^ (Fig. 1, *C* and *G*). These findings were further validated by the use of isolated brain and heart mitochondria isolated from MCU KO mice and studied in the presence and absence of RR (Fig. 1, *B*, *D*, *F*, and *H*). A caveat of our approach, however, was that all experiments were performed at high pyruvate concentrations. We thus set up pyruvate titration experiments using isolated brain and heart mitochondria under phosphorylating (state 3) conditions at low (∼12 nm), moderately elevated (800 nm) Ca^2+^ concentrations as well as in the additional presence of RR (Fig. 2, *A* and *B* (brain) and *C* and *D* (heart)). Our experiments revealed a substantial pyruvate dependence of respiratory rates. However, no difference was observed between the shape of the titration curves, indicating similar kinetic constants irrespective of the presence and/or absence of MCU activity. These results argue for a high basal, non-Ca^2+^-dependent, PDH activity, which makes OXPHOS regulation across broad physiologic ranges of workload through Ca^2+^ stimulation of mitochondrial pyruvate oxidation an unlikely condition. Conversely, our data indicate that even small changes within the range of physiological pyruvate concentrations of ∼175 μm in the brain (14) can easily adapt OXPHOS rates (Fig. 2, *A–D*). We conclude that MCU activity, and thus the presence of mitochondrial matrix Ca^2+^, is largely dispensable for OXPHOS activation when driven by PDH-dependent pyruvate oxidation but is required for stimulation of OXPHOS activity beyond 85% of *V*_max_.

### Glutamate oxidation is stimulated by extramitochondrial Ca^2+^

**Figure 3.**
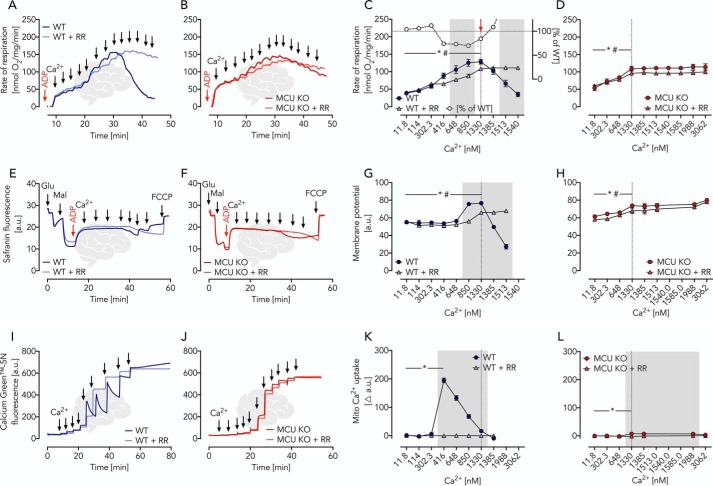
**Ca^2+^-dependent glutamate/malate-driven OXPHOS in brain mitochondria isolated from WT and MCU KO mice characterized by parallel measurements of respiratory rates, membrane potential, and Ca^2+^ uptake.** *A* and *B*, respiratory traces of WT and MCU KO brain mitochondria in EGTA media supplemented with glutamate, malate, ADP, and RR as indicated; *arrows* specify Ca^2+^ additions for a stepwise increase from ∼12 nm to 2 μm. *C*, respiratory rates of WT brain mitochondria in the presence and absence of RR as indicated shown as mean ± S.E. (*error bars*) of *n* = 5 experiments plotted against free Ca^2+^ concentrations. The *red arrow* indicates significantly increased Ca^2+^-induced respiration in WT (*, *p* < 0.05) and WT + RR (#, *p* < 0.05) mitochondria determined using two-way ANOVA with Dunnett's multiple-comparison post hoc test. *Gray area*, significant difference between WT and WT + RR mitochondria determined using two-way ANOVA analysis with Sidak's multiple-comparison post hoc test and a *p* value of <0.05. *White circles*, percentage of WT + RR respiration compared with WT calculated from the mean values. *D*, respiratory rates of MCU KO brain mitochondria in the presence and absence of RR as indicated shown as mean ± S.E. of *n* = 5 experiments plotted against free Ca^2+^ concentrations. Statistical analyses were done as described for *C*. *, *p* < 0.05 in MCU KO. #, *p* < 0.05 in MCU KO + RR. *E* and *F*, mitochondrial membrane potential traces of WT and MCU KO brain mitochondria using safranin O as probe supplemented with glutamate, malate, ADP, and RR as indicated; *a.u.*, arbitrary units; fluorescence after FCCP uncoupling served as zero value; *arrows* indicate Ca^2+^ additions of 100 μm each for a stepwise increase from ∼12 nm to 5 μm. *G* and *H*, mitochondrial membrane potential shown as mean ± S.E. of *n* = 5 experiments plotted against free Ca^2+^ concentrations. Statistical analyses were done as described for *C* and *D. I* and *J*, Ca^2+^ uptake traces in WT and MCU KO brain mitochondria using Calcium Green^TM^-5N as a probe supplemented with glutamate, malate, ADP, and RR; *arrows* indicate Ca^2+^ additions for a stepwise increase from ∼12 nm to 2 μm. *K* and *L*, changes in Ca^2+^ uptake shown as mean ± S.E. of *n* = 5 experiments plotted against free Ca^2+^ concentration. Statistical analyses were done as described for *C* and *D*.

We next tested glutamate-malate as a substrate combination. Of note, there are at least two different types of mitochondrial glutamate transporters (29), namely (i) Ca^2+^-independent glutamate carriers (30), GC1 and GC2, and (ii) Ca^2+^-dependent mitochondrial aspartate-glutamate carriers, Aralar and Citrin (19, 23–25). Whereas GC1 and GC2 import glutamate mainly for its degradation, forming α-oxoglutarate (α-OG) and ammonia, the activity of the Ca^2+^-dependent mitochondrial aspartate-glutamate carrier, Aralar, depends on the formation of aspartate (see Fig. 5*A*) through transamination and oxaloacetate formation facilitated by the malate dehydrogenase (MDH) as well as the exchange of malate against α-OG. Therefore, the Aralar-dependent pathway involves and represents the activity of the mitochondrial part of the MAS. This allowed the set-up of experiments specifically addressing the Ca^2+^ dependence of the mitochondrial glutamate metabolism without the pyruvate metabolization (minimal MAS, Table 1, mode 1) (19, 23–25). In the presence of ADP (state 3) and absence of Ca^2+^, the measured glutamate metabolism of brain mitochondria was low in WT (Fig. 3*A*). A stepwise increase of Ca^2+^ revealed, in contrast to pyruvate oxidation, a three-phase respiratory response (Fig. 3). In phase 1 at Ca^2+^ concentrations less than 400 nm, we observed a small but significant stimulation of respiration (Fig. 3, *A* and *C*), whereas the mitochondrial membrane potential remained unaffected (Fig. 3, *E* and *G*) and Ca^2+^ was not taken up (Fig. 3, *I* and *K*). In phase 2 at Ca^2+^ concentrations above 400 nm, we observed an increase of both mitochondrial respiration (Fig. 3, *A* and *C*) and membrane potential (Fig. 3, *E* and *G*) as well as mitochondrial Ca^2+^ uptake (Fig. 3, *I* and *K*). The detected mitochondrial Ca^2+^ uptake may account for the increase in respiratory rates (Fig. 3*C*) through the activation of intramitochondrial dehydrogenases, such as α-oxoglutarate dehydrogenase and isocitrate dehydrogenase. This notion is supported by an observed RR sensitivity. However, our data also indicate that the respiratory activation depends to a great extent on extramitochondrial Ca^2+^ (respiration in percentage of WT, Fig. 3*C*). In phase 3 at high concentrations of Ca^2+^ (>1.3 μm), we observed decreased respiratory rates (Fig. 3, *A* and *C*) and membrane potential (Fig. 3, *E* and *G*) in WT mitochondria. The latter showed mitochondrial Ca^2+^ efflux (Fig. 3, *I* and *K*). This phenomenon is known as mitochondrial Ca^2+^ overload toxicity and is fully abolished by the addition of RR. This finding is unlikely to be a substrate-specific artifact because similar effects were also seen for other substrate combinations (*e.g.* pyruvate-malate-driven measurements of the mitochondrial membrane potential) (Fig. S5, *A* and *B*). Using isolated MCU KO brain mitochondria, we observed a similar Ca^2+^-dependent stimulation of respiration and membrane potential (Fig. 3, *B*, *D*, *F*, and *H*) with a very small but significant RR-sensitive Ca^2+^ uptake at concentrations above 1.5 μm (Fig. 3 (*J* and *L*) and Fig. S1*D*). This RR-sensitive Ca^2+^ uptake affected respiratory rates (Fig. 3, *B* and *D*) and mitochondrial membrane potential (Fig. 3, *F* and *H*) and may be attributable to MCU activity based on residual *Mcu* transcript expression (Fig. S2, *A–C*) or other nonspecific side effects of RR.

**Table 1:** **Description of different levels of stepwise MAS reconstitution (relates to Figs. 3–8)**

	Stepwise MAS reconstitution	Constituents	Metabolic consequences and use of inhibitors/effectors
Mitochondria	Mode 1 (minimal MAS)	Glutamate-malate, ADP	Glutamate-malate-driven respiration (state 3) controlled by extramitochondrial Ca^2+^ through activation of Aralar.
	Mode 2 (incomplete MAS)	Glutamate-malate, ADP + α-oxoglutarate, aspartate, lactate, NADH	MAS substrates present but in the absence of MAS enzymes; this mode lacks the MAS-driven generation of pyruvate and is thus not yet fully functional but extramitochondrial Ca^2+^-sensitive.
	Mode 3 (complete reconstituted MAS)	Glutamate-malate, ADP, α-oxoglutarate, aspartate, lactate, NADH + LDH, GOT, and MDH	MAS substrates and enzymes added, thus allowing a continuous generation of pyruvate at the maximum rate, depending on extramitochondrial Ca^2+^ level. Cin inhibits mitochondrial pyruvate uptake and AOA inhibits MAS enzyme GOT (both inducing pyruvate starvation).
Cells	Mode 4 (complete endogenous MAS)	All constituents endogenously present as described for MAS mode 3 but in their natural stoichiometries	Endogenous MAS activity controlled by cytosolic Ca^2+^. AOA inhibits MAS enzyme GOT and BAPTA-AM chelates Ca^2+^ to restrict MAS activation (both inducing a state of pyruvate starvation).
Heart	Mode 5 (complete endogenous MAS)	All constituents endogenously present as described for MAS mode 3 but in their natural stoichiometries	Endogenous MAS activity controlled by cytosolic Ca^2+^; AOA inhibits MAS enzyme GOT (inducing a state of pyruvate starvation).

**Figure 4.**
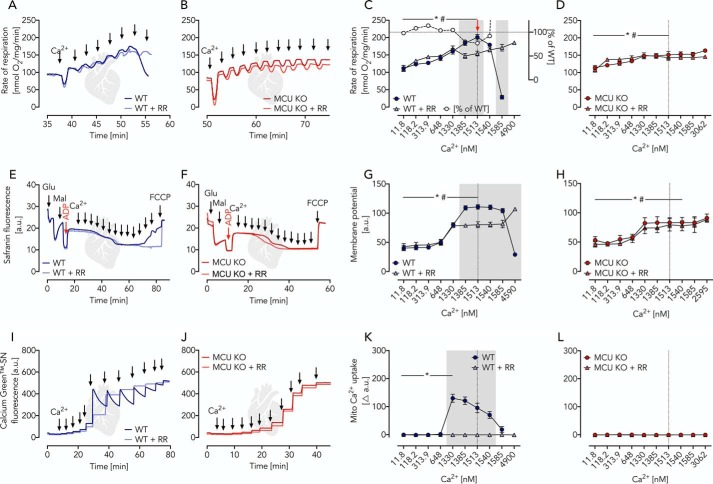
**Ca^2+^-dependent glutamate/malate-driven OXPHOS in heart mitochondria isolated from WT and MCU KO mice characterized by parallel measurements of respiratory rates, membrane potential, and Ca^2+^ uptake under similar conditions.** *A* and *B*, respiratory traces of WT and MCU KO heart mitochondria in EGTA media supplemented with glutamate, malate, ADP, and RR as indicated; *arrows* specify Ca^2+^ additions for a stepwise increase from ∼12 nm to 2 μm. *C*, respiratory rates of WT heart mitochondria in the presence and absence of RR as indicated shown as mean ± S.E. (*error bars*) of *n* = 5 experiments plotted against free Ca^2+^ concentrations. The *red arrow* indicates significantly increased Ca^2+^-induced respiration in WT (*, *p* < 0.05) and WT + RR (#, *p* < 0.05) mitochondria determined using two-way ANOVA with Dunnett's multiple-comparison post hoc test. *Gray area*, significant difference between WT and WT + RR mitochondria determined using two-way ANOVA with Sidak's multiple-comparison post-hoc test and a *p* value of <0.05. *White circles* visualize the percentage of WT + RR respiration compared with WT calculated from the mean values. *D*, respiratory rates of MCU KO heart mitochondria in the presence and absence of RR as indicated shown as mean ± S.E. of *n* = 5 experiments plotted against free Ca^2+^ concentrations. Statistical analyses were done as described for *C*. *, *p* < 0.05 in MCU KO; #, *p* < 0.05 in MCU KO + RR. *E* and *F*, mitochondrial membrane potential traces of WT and MCU KO heart mitochondria using safranin O as probe supplemented with glutamate, malate, ADP, and RR as indicated; *a.u.*, arbitrary units; fluorescence after FCCP uncoupling served as zero value; *arrows* indicate Ca^2+^ additions of 100 μm each for a stepwise increase from ∼12 nm to 5 μm. G and H, mitochondrial membrane potential shown as mean ± S.E. of *n* = 5 experiments plotted against free Ca^2+^ concentrations. Statistical analyses were done as described for *C* and *D. I* and *J*, Ca^2+^ uptake traces in WT and MCU KO heart mitochondria using Calcium Green^TM^-5N as probe supplemented with glutamate, malate, ADP, and RR; *arrows* indicate Ca^2+^ additions for a stepwise increase from ∼12 nm to 2 μm. *K* and *L*, changes in Ca^2+^ uptake shown as mean ± S.E. of *n* = 5 experiments plotted against free Ca^2+^ concentration. Statistical analyses were done as described for *C* and *D*.

**Figure 5.**
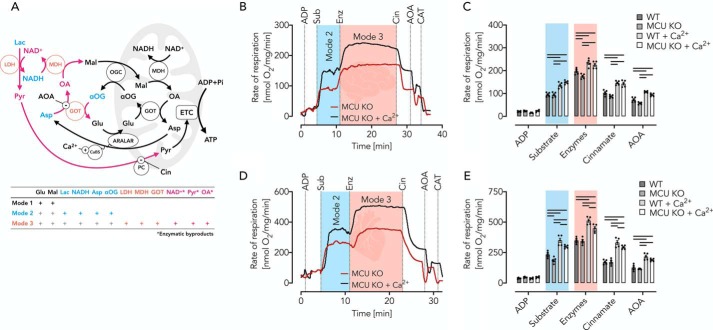
**Ca^2+^-dependent respiration of brain and heart mitochondria isolated from WT and MCU KO mice fueled by reconstituted complete MAS.** *A*, *schematic* of MAS with modes demonstrating the increasing complexity of the different experimental modes used according to Table 1. Mode 1 refers to incubations containing glutamate and malate (*black*). In mode 2, incubations contain in addition lactate (*Lac*), NADH, Asp, and α-OG (*blue*). In mode 3, incubations contain in addition the enzymes LDH, MDH, and GOT (*red*), allowing the formation of NAD^+^, pyruvate (*Pyr*), and oxaloacetate (*OA*) (*pink*). Cin is the inhibitor of the mitochondrial PC; AOA is the GOT inhibitor. *B*, respiratory traces of MCU KO brain mitochondria (0.06 mg/ml) in EGTA media in the presence or absence of 800 nm Ca^2+^, supplemented with malate, NADH, and lactate. Measurements were started by the ADP addition (2 mm). The addition of the substrates (*Sub*) glutamate, aspartate, and α-oxoglutarate adjusts mode 2. The addition of the enzymes (*Enz*) LDH, MDH, and GOT adjusts mode 3. Cin and AOA allow the determination of the pyruvate– and hydride anion–dependent contribution of the complete MAS to substrate supply for OXPHOS. Carboxyatractyloside (*CAT*) indicates the presence of phosphorylating respiration. *C*, respiratory rates of WT and MCU KO brain mitochondria shown as mean ± S.E. (*error bars*) of *n* = 5 experiments. *Horizontal bars*, *p* < 0.05 for comparison as indicated, determined using two-way ANOVA with Tukey's multiple-comparison test. *D*, respiratory traces of MCU KO heart mitochondria (0.04 mg/ml) under conditions as described in *B. E*, respiratory rates of WT and MCU KO heart mitochondria shown as mean ± S.E. of *n* = 5 experiments. *Horizontal bars*, *p* < 0.05 for comparison as indicated, determined using two-way ANOVA with Tukey's multiple-comparison test.

Isolated heart mitochondria revealed a very similar behavior, essentially replicating the above experiments in brain mitochondria. In contrast to brain mitochondria, however, the Ca^2+^-insensitive glutamate-malate–activated respiration of heart mitochondria was 2-fold larger but decreased significantly in the course of 30 min of preincubation (Fig. S3*C*). Ca^2+^ titration upon preincubation revealed Ca^2+^ responses comparable with those seen for brain mitochondria (Fig. 4, *A–L*). Taken together, the Ca^2+^-dependent glutamate-malate–controlled OXPHOS in heart and brain mitochondria with stimulations up to 200–300% represents the minimal version of the MAS (Table 1, mode 1) whereby glutamate uptake is facilitated through a Ca^2+^-dependent pathway facilitated by Aralar (Fig. 5*A*). The lack of further MAS substrates and enzymes (restricted complexity), however, limits full MAS capacity. Furthermore, in mitochondria of both origins (brain and heart), a Ca^2+^-independent glutamate-driven OXPHOS can be measured (brain < heart).

### MAS reconstitution boosts cytosolic pyruvate supply for OXPHOS

To study the true impact of the MAS on pyruvate supply for OXPHOS activation, we reconstituted the MAS in a stepwise manner *in vitro* and *ex vivo* to reach a system of continuous (steady-state) generation of pyruvate from lactate. Together with the use of specific inhibitors and Ca^2+^ chelators, this allowed us to unambiguously separate the role of cytosolic from mitochondrial matrix Ca^2+^ (Table 1 (modes 1–5) and Fig. 5*A*).

In a first set of experiments, we used brain mitochondria isolated from WT and MCU KO mice (Fig. 5, *B* and *C*). The MAS reconstitution assay was done in EGTA media (to adjust different Ca^2+^ levels) supplemented with the nonfeeding substrates malate, lactate, and NADH. Expectedly, the addition of 2 mm ADP had only minimal effects on respiratory rates (Fig. 5*B*). Next, the addition of glutamate, α-oxoglutarate, and aspartate adjusted MAS mode 2 (incomplete MAS, Table 1 and Fig. 5*A*). At this mode, we detected a Ca^2+^-sensitive OXPHOS stimulation, which was limited by the lack of extramitochondrial pyruvate formation (Fig. 5, *A–C*). In support of this assumption, the addition of MAS enzymes (lactate dehydrogenase (LDH), glutamate oxaloacetate transaminase (GOT), and MDH, complete reconstituted MAS, Table 1, mode 3) induced a substantial stimulation of OXPHOS in a Ca^2+^-sensitive manner (Fig. 5, *A–C*). To demonstrate that the observed OXPHOS stimulation was due to MAS-mediated extramitochondrial pyruvate generation, we added cinnamate (Cin), a specific inhibitor of the mitochondrial pyruvate carrier (PC) and observed a marked OXPHOS inhibition (Fig. 5, *B* and *C*). This inhibition was not complete, however, because Cin does not impair MAS activity. Thus, mitochondrial hydride anion uptake (Fig. 5*A*) remained unaffected, allowing residual respiration. To confirm this assumption, we next inhibited the transport of hydride anions using the GOT inhibitor aminooxyacetate (AOA), which further decreased OXPHOS activities. The degree of activation of OXPHOS rates controlled by extramitochondrial Ca^2+^ was comparable in WT and MCU KO mitochondria (Fig. 5, *B* and *C*). This indicates that the experimental elevation of OXPHOS rates was largely independent from MCU activities. Moreover, and most importantly, we demonstrate that extramitochondrial pyruvate generation and supply to mitochondria in a Ca^2+^-sensitive manner may be a major task of the MAS (Fig. 5, *A–C*). This puts the MAS at center stage of metabolic homeostasis. Using isolated heart mitochondria, we confirmed the above findings (Fig. 5, *D* and *E*), albeit revealing an almost double capacity of MAS-controlled pyruvate generation in the heart as compared with brain mitochondria (Fig. 5, *B–E*).

### Cytosolic Ca^2+^ controls pyruvate generation in synaptosomes and primary cells

**Figure 6.**
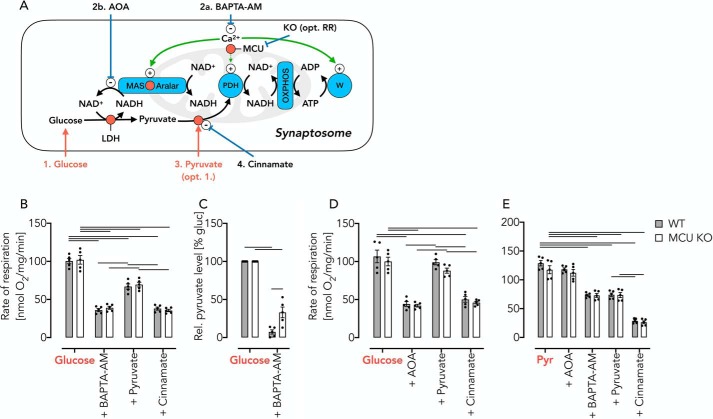
**Detection of reversible pyruvate starvation after AOA– or BAPTA-AM–induced inhibition of the endogenous MAS in intact synaptosomes.** *A*, *schematic* of experimental set-up. The *numbers* indicate the order of addition. *B*, respiratory rates of WT and MCU KO synaptosomes in KRB supplemented with glucose/lactate, 20 min after the addition of BAPTA-AM (50 μm), after the addition of pyruvate (10 mm), and after inhibition of the mitochondrial pyruvate carrier using Cin. Of note, BAPTA-AM–induced Ca^2+^ depletion affects both the cellular workload and the substrate supply for OXPHOS. *C*, pyruvate levels in incubations of synaptosomes with glucose (10 mm)/lactate (10 mm) before and after the addition of BAPTA-AM (50 μm). *D*, respiratory rates in WT and MCU KO synaptosomes using AOA instead of BAPTA-AM to induce a state of pyruvate starvation. *E*, respiratory rates in WT and MCU KO synaptosomes measured in KRB supplemented with pyruvate (*Pyr*) under the conditions indicated. Here, BAPTA-AM–induced Ca^2+^-depletion affects workload but *not* mitochondrial substrate supply. All data are shown as mean ± S.E. (*error bars*) of *n* = 5 experiments. *Horizontal bars* in *B*, *D*, and *E*, *p* < 0.05 for comparison as indicated, determined using two-way ANOVA with Tukey's multiple-comparison test. *Horizontal bars* in *C*, *p* < 0.05 for comparison as indicated, determined using two-way ANOVA with Sidak's multiple-comparison post hoc test.

**Figure 7.**
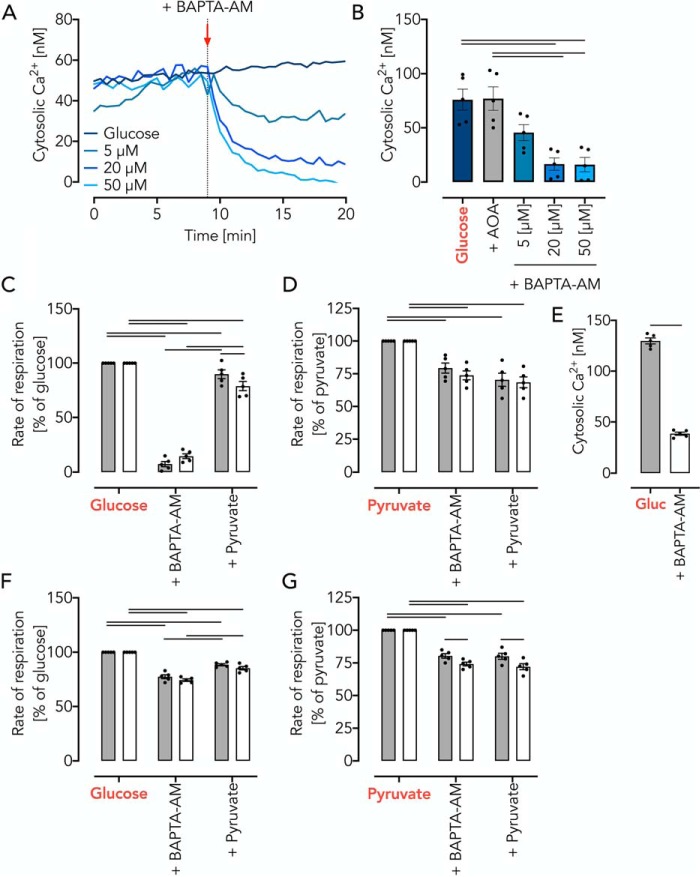
**Mitochondrial pyruvate supply is controlled by cytosolic Ca^2+^.** *A*, BAPTA-AM decreases cytosolic Ca^2+^ in a concentration-dependent manner as measured by Fura-2/AM in synaptosomes incubated in KRB supplemented with glucose (10 mm)/lactate (10 mm). *B*, cytosolic Ca^2+^ levels in glucose/lactate-fed synaptosomes treated with increasing concentrations of BAPTA-AM or AOA. Data are shown as mean ± S.E. (*error bars*) of *n* = 5 experiments. *Horizontal bars*, significant difference with *p* < 0.05 for comparison as indicated, determined using one-way ANOVA with Tukey's multiple-comparison test. test. *C*, respiratory rates of glucose (10 mm)/lactate (10 mm)-fed thymocytes, 20 min after the addition BAPTA-AM (50 μm), and after addition of pyruvate (10 mm). Data are shown as mean ± S.E. (in percentage of glucose/lactate respiration) of *n* = 5 experiments. *Horizontal bars*, significant difference with *p* < 0.05 determined using two-way ANOVA with Tukey's multiple-comparison test for comparisons within the WT or MCU KO groups and Sidak's multiple-comparison post hoc test for comparisons between the WT and MCU KO groups. *D*, respiratory rates of pyruvate (10 mm) fed thymocytes, 20 min after the addition BAPTA-AM (50 μm), and after addition of pyruvate (10 mm). Data are shown as mean ± S.E. of *n* = 5 experiments. *Horizontal bars*, significant difference with *p* < 0.05 determined using two-way ANOVA with Tukey's multiple-comparison test for comparisons as indicated. *E*, free Ca^2+^ levels measured using Fura-2/AM in glucose (10 mm)/lactate (Gluc; 10 mm)-fed MCU KO thymocytes and 20 min after treatment with BAPTA-AM (50 μm). Data are shown as mean ± S.E. of *n* = 5 experiments. *Horizontal bars*, significant difference with *p* < 0.05 determined using Student's *t* test. *F*, respiratory rates of glucose-fed (*Gluc*; 20 mm) fibroblasts with sequential additions of BAPTA-AM (50 μm) and pyruvate (10 mm). Data are shown as mean ± S.E. (in percentage of pyruvate respiration) of *n* = 5 experiments. *Horizontal bars*, significant difference with *p* < 0.05 determined using two-way ANOVA with Tukey's multiple-comparison test for comparisons as indicated. *G*, respiratory rates of pyruvate-fed fibroblasts with sequential additions of BAPTA-AM (50 μm) and pyruvate (10 mm). Data are shown as mean ± S.E. of *n* = 5 experiments. *Horizontal bars*, significant difference (*p* < 0.05) determined using two-way ANOVA with Tukey's multiple-comparison test for comparisons within the WT or MCU KO groups and Sidak's multiple-comparison post hoc test for comparisons between the WT and MCU KO groups.

We next aimed to test whether MAS-stimulated OXPHOS control is equally important within intact cells. This was done in isolated synaptosomes, thymocytes, and fibroblasts from WT and MCU KO mice. We first used intact synaptosomes, which are specifically suited to study cellular energy metabolism (31). Mode 4 (complete endogenous MAS, Table 1) was adjusted by feeding synaptosomes with glucose/lactate, allowing substantial OXPHOS rates in the presence of physiologic (endogenous) Ca^2+^ concentrations (Fig. 6*A*). Chelation of cytosolic Ca^2+^ using membrane-permeable BAPTA-AM decreased free Ca^2+^ (Fig. 7, *A* and *B*) and concomitantly restricted OXPHOS activity (Fig. 6*B*). Because cytosolic Ca^2+^ is required for the generation of pyruvate in the cytosol, BAPTA-AM indeed induced a condition of mitochondrial pyruvate starvation (Fig. 6, *A* and *C*). Pyruvate supplementation partially restored OXPHOS activity in a MAS-independent manner (Fig. 6*B*) despite the BAPTA-AM–mediated decrease of cytosolic Ca^2+^. To demonstrate that OXPHOS restoration was indeed based on replenished pyruvate level, we prevented its mitochondrial uptake using Cin (Fig. 6*B*), which decreased OXPHOS activity to levels seen prior to pyruvate supplementation (Fig. 6*B*). As an independent control, we repeated the experiments but inhibited MAS activity using AOA (32). Again, we observed a decrease of OXPHOS activity (Fig. 6*D*) to levels previously seen in the presence of BAPTA-AM (Fig. 6*B*). We hypothesized that in the presence of AOA again a state of pyruvate starvation was induced despite high cytosolic Ca^2+^ concentrations, and indeed, pyruvate replenishment recovered OXPHOS activity nearly completely in a Cin-sensitive manner (Fig. 6*D*). This demonstrates that AOA, similar to BAPTA-AM, inhibits the MAS activity but leaves cellular workload unaffected. Specifically, MAS activation by cytosolic Ca^2+^ is essential to generate a sufficient amount of pyruvate for OXPHOS in intact synaptosomes. This notion was further substantiated by feeding synaptosomes with pyruvate instead of glucose/lactate (Fig. 6*E*). Under such conditions, decreased cytosolic Ca^2+^ levels have no effect on mitochondrial pyruvate supply despite a presumed decrease of cellular workload. Indeed, further pyruvate addition failed to reactivate the respiratory rate while Cin inhibited respiration.

We replicated all of the above experiments using intact primary thymocytes and fibroblasts, essentially giving the same results (Fig. 7, *C–G*). From the data, we concluded that mitochondrial pyruvate oxidation rates are not controlled by mitochondrial matrix Ca^2+^. Instead, cytosolic Ca^2+^ levels control pyruvate generation through reversible activation of the MAS.

### Cytosolic pyruvate generation by MAS is required for cardiac power

**Figure 8.**
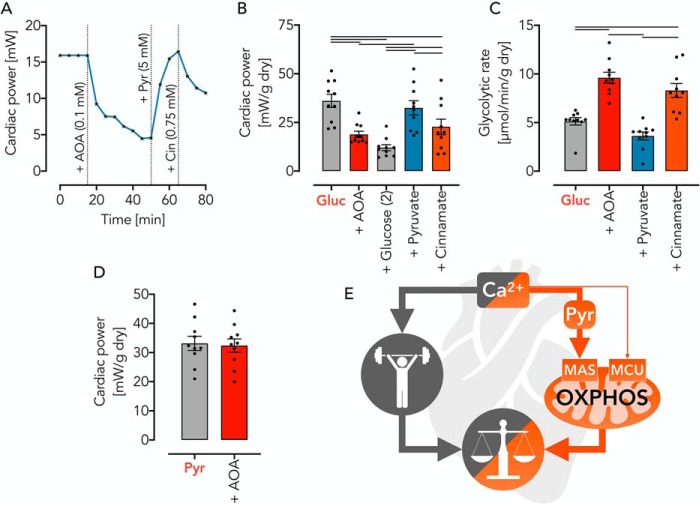
**Pyruvate supply by the MAS controls cardiac power in the working rat heart *ex vivo*.** *A*, representative pressure response trace during isolated heart perfusion with substrates (glucose (*Gluc*) and pyruvate (*Pyr*)) and inhibitors (AOA and Cin) added as indicated. *B*, cardiac power under metabolic conditions as indicated. *C*, glycolytic rate under conditions as indicated. Note that there is a reverse relationship of cardiac power and glycolytic rates, depending on metabolic conditions. *D*, cardiac power in isolated rat hearts using pyruvate as substrate. Note that AOA-induced inhibition of MAS does not affect the use of pyruvate. All data are shown as mean ± S.E. (*error bars*) of *n* = 10 experiments. *Horizontal bars*, significant difference with *p* < 0.05 determined using two-way ANOVA with Tukey's multiple-comparison test for comparisons as indicated. *E*, graphical abstract summarizing Ca^2+^-controlled balance of cellular workload and mitochondrial OXPHOS. Cytosolic pyruvate generation allows OXPHOS activation to a great extent, depending on demand and organ, whereas the mitochondrial Ca^2+^ import through MCU is, under physiologic conditions, largely dispensable.

To resolve the question whether MAS activity controls OXPHOS also in whole organs, we studied cardiac contractility and metabolism in the perfused isolated working rat heart (complete endogenous MAS, Table 1, mode 5, Fig. 5*A*). We found that cardiac power was significantly diminished upon AOA inhibition of the MAS (Fig. 8, *A* and *B*). The fact that glucose addition did not overcome AOA-mediated inhibition of cardiac power (Fig. 8*B*) indicates that the underlying mechanism was not based on glucose starvation.

In contrast, an observed decrease in cardiac power was specific to MAS inhibition, evidenced by a complete contractile restoration upon pyruvate replenishment (Fig. 8, *A* and *B*). In support of this notion, inhibition of mitochondrial pyruvate uptake by Cin impaired cardiac power (Fig. 8, *A* and *B*). Equally important is the observation that hearts with a pharmacologically impaired MAS and/or pyruvate uptake inhibition showed a reciprocal increase in glycolysis (Fig. 8*C*). This indicates that cardiac power and metabolism are intimately interrelated with MAS, generating sufficient amounts of pyruvate for OXPHOS (Fig. 8, *D* and *E*). To test this idea, we measured cardiac power in isolated rat hearts using pyruvate as substrate. With pyruvate as substrate, AOA failed to decrease the baseline power (Fig. 8*D*), once again excluding a MAS-unrelated toxic effect of AOA under such conditions. Unlike in isolated mitochondria and intact synaptosomes and primary cells, we used in the heart only AOA and not BAPTA-AM to test our hypothesis because the expected slow uptake of BAPTA-AM impedes its use in the working mammalian heart (33).

## Discussion

In the present study, we addressed a fundamental question of bioenergetics (*i.e.* how Ca^2+^ balances mitochondrial OXPHOS activity and the rate of ATP-splitting reactions, thereby avoiding both substrate limitations and overenergizations). Current models assume that Ca^2+^-mediated activation of mitochondrial matrix dehydrogenases controls the substrate supply for OXPHOS with mitochondrial Ca^2+^ uptake being facilitated by MCU. Although Ca^2+^-mediated activation of matrix pyruvate dehydrogenase in mitochondria is beyond doubt (4, 9), an interrelated direct activation of pyruvate-driven OXPHOS flux still lacks proper experimental evidence. Indeed, it was previously shown that under physiologic conditions, an activation of PDH can be detected by enzymatic methods without finding a correlate in respiratory activation (4). This paradox may exist because in WT mitochondria in the absence of Ca^2+^ and MCU KO mitochondria, PDH is enzymatically sufficiently active to allow maximal OXPHOS flux. Therefore, an increase of mitochondrial Ca^2+^ may further increase the PDH enzymatic activity, but this has no measurable impact on pyruvate-dependent respiration. Starvation represents one exception to this rule because it induces the phosphorylation of regulatory PDH subunits and thereby decreases its enzymatic activity (4). Thus, only when the maximal OXPHOS flux is restricted by the enzymatic PDH activity, such as seen under starvation, does the Ca^2+^ dependence of pyruvate-driven mitochondrial respiration become detectable (4, 9). Nevertheless, maximal rates of Ca^2+^-stimulated pyruvate-dependent respiration under all circumstances reach comparable values (9, 16). From these data, we conclude that the mitochondrial rate of pyruvate oxidation cannot be controlled by a mechanism that requires mitochondrial Ca^2+^ uptake. Instead, we describe that for up to 85% of OXPHOS *V*_max_, the function of the MCU and activation of mitochondrial matrix dehydrogenases are dispensable (graphical overview in Fig. 8*E*). We propose an alternative mechanism based on the activity of the MAS, which controls cytosolic pyruvate generation and its mitochondrial supply.

Of note, the MCU in brain mitochondria requires Ca^2+^ concentrations of >400 nm for catalytic activity, whereas the effects described in this study are seen at lower (presumably more physiologic) levels (34, 35). Conversely, our pyruvate titration experiments revealed that concentrations of 175 μm pyruvate, as detected in the neuronal cytosol (14), are within the control area of pyruvate-dependent respiration. This demonstrates that mitochondrial OXPHOS activity follows changes in the (cytosolic) pyruvate supply. Furthermore, our data suggest that a MAS-dependent activation of OXPHOS exists in many tissues and cells because we were able to identify it to different degrees in isolated mitochondria from mouse brain and heart; mouse synaptosomes, thymocytes, and fibroblast; and, most importantly, in the working rat heart. The use of the different models from isolated mitochondria (low biological complexity) to the working rat heart (high biological complexity) in combination with inhibitors and effectors to reconstitute the complete MAS in a stepwise manner as well as the use of WT and MCU KO mice allowed us to disentangle causes and effects of Ca^2+^ signaling from different cellular compartments on OXPHOS activity. Specifically, the importance of the Ca^2+^-sensitive MAS component, Aralar, on mitochondrial pyruvate supply was previously demonstrated in mouse Aralar KO neurons (26). In humans, Aralar deficiency causes a neurodevelopmental disorder with severe impairment of neuronal respiration (36, 37), and Aralar KO in the mouse recapitulates the symptoms to a great extent (38). The fact that Aralar KO mice do not survive beyond 19–21 days further substantiates its biological importance for the cellular energy metabolism.

It is worth mentioning, however, that many cell types, including neurons, astrocytes, and white skeletal muscles, but not cardiomyocytes, express in addition to MAS the glycero-3-phosphate shuttle (24, 39) as a second system, which allows pyruvate formation by oxidizing cytosolic NADH. Also noteworthy is the fact that other tissues, such as brown adipose tissue, lack the MAS (24) or have an additional pathway to oxidize cytosolic NADH, the NADH cytochrome *c* oxidoreductase, for instance described for liver tissue (40). The complex interplay between the different Ca^2+^-regulated systems and their individual or combinational significance in different organs for the development of human pathologies remains to be elucidated in future studies. It will be interesting to elucidate whether the different pathways can compensate for each other under different pathologic conditions and why these pathways are cell type–specific despite having similar biological functions.

Interestingly, different pathologies based on dysfunction of MCU (and related proteins) exist (41–43). One obvious explanation for the lack of a clear-cut phenotype in MCU KO mice may be that the animals are maintained under metabolically unstressed conditions. Indeed, different conditions and even a change in genetic background impact the MCU KO mouse phenotype (18). With the exception of body weight and swim velocity, we found that MCU KO mice appear nearly indistinguishable from WT controls, including their behavioral responses (open field and Morris water maze tests). Others detected diminished muscle performance at high energy demands (9, 12), confirming a function of MCU for fight-and-flight reactions (12), which is irrelevant for the conditions tested here. Nevertheless, this supports our notion that MCU is dispensable for OXPHOS control for a broad range of physiologic conditions.

Taken together, a MAS-mediated pyruvate supply exists in a number of cells and tissues and controls OXPHOS under physiologic conditions to a great extent. The conceptional and pathophysiologic consequences of our findings are of broad importance, considering that a better understanding of mitochondrial Ca^2+^ handling is essential to understand OXPHOS control mechanisms and metabolic homeostasis. Moreover, our findings may help to develop new models to tackle diseases in which cell death is initiated by mitochondrial Ca^2+^ overload, a pathophysiological mechanism underlying a number of neurodegenerative diseases and responsible for post-ischemic heart injuries.

## Experimental procedures

### Animals

MCU KO mice (IST11669F8 gene trap insertion into intron 1 of the *Mcu* gene) (9) and WT controls were obtained from the Texas Institute for Genomic Medicine. MCU KO mice were kept on a CD1-ICR background (>96%) after backcrossing from C57BL/6 for a minimum of 6 generations. Sprague–Dawley rats were bred at the animal facility of the Jena University Hospital and originally derived from Janvier Laboratories (La Rochelle, France). All experiments were performed in accordance with the ethical guidelines for animals in experiments and were approved by the local animal care committees (Landesverwaltungsamt Sachsen-Anhalt, Germany, permit ID IPHY/G/01-1383/16; Landesamt für Verbraucherschutz Thüringen, Germany, permit ID TWZ-15-2018).

### RNA isolation and complementary DNA synthesis

Hippocampal and heart total RNA was isolated by disrupting and homogenizing tissues in TRIzol reagent (Thermo Fisher Scientific, catalog no. 15596018) and RNA purification using the RNeasy Mini Kit (Qiagen, catalog no. 74104). Complementary DNA was transcribed from template RNA using random primers and the iScript Select cDNA Synthesis Kit (Bio-Rad, catalog no. 1708897).

### Quantitative PCR

Gene expression was measured on an iQ5 qPCR system (Bio-Rad) using SYBR Green qPCR master mix (Bimake, catalog no. B21202) or iTaq Universal Probes Supermix (Bio-Rad, catalog no. 1725130). *CT* values were defined at the inflection points of fitted sigmoid curves (four-parameter Chapman curves) and were compared with mitochondrial processing peptidase (*Pmpca*).

### Oligonucleotide primers used for PCR

Primers used were as follows: *Mcu* forward, 5′-GTA CGG CCA CCA AAG AGA GAC CTC-3′; *Mcu* reverse, 5′-ACA GCA CCA GAG TGG TCC TCT-3′; TaqMan probe, 5′-6-carboxyfluorescein-TAA GCC ATG AAG ATG CAG CGA CGC TGA A-*N*,*N*,*N*′,*N*′-tetramethyl-6-carboxyrhodamine-3′; *Pmpca* forward, 5′-GTG ACT GCC AGA CCT CAA GAG ACA C-3′; *Pmpca* reverse, 5′-GAT CGA TCT TCG CTA TGT TCT CTA CAG-3′.

### Open field test

Spontaneous motor activity was assessed as described (44). To test for anxiety behavior, a virtual center area (30 × 30 cm^2^) was defined, and the time spent in this area was measured as proxy for absence of anxiety.

### Morris water maze test

The Morris water maze test was performed as described (45). Briefly, animals were trained on 4 consecutive days with 4 training trials/day. Each trial ended when the animal reached the platform or after 60 s. On day 5, the submerged platform was removed. Memory performance was measured as the time until the target quadrant was reached.

### Isolation of mitochondria

Brain mitochondria were isolated as described (19, 46). To ensure comparable conditions with respect to mitochondrial Ca^2+^ content, heart mitochondria were prepared following the same protocol but in the absence of digitonin.

### Ca^2+^ measurements

Mitochondrial Ca^2+^ uptake was measured using a Cary Eclipse fluorescence spectrophotometer (Agilent) and Calcium Green^TM^-5N as fluorescent probe (Invitrogen Molecular Probes, catalog no. C3737; 0.5 μm) at 506/532 nm excitation/emission wavelengths, respectively. Measurements were done in EGTA media (Figs. 3 (*I* and *J*) and 4 (*I* and *J*)) or in EGTA-free media (Fig. S1 (*A*, *B*, *E*, and *F*)). Free extramitochondrial Ca^2+^ concentrations in EGTA-containing buffers were measured using Fura-2 (Thermo Fisher Scientific, catalog no. F1200; 10 μm) at 340 and 380/510 nm excitation/emission wavelengths, respectively. For these measurements, mitochondria were incubated in EGTA media under conditions used for respirometry. Cytosolic Ca^2+^ concentrations in fibroblasts, thymocytes, or synaptosomes were measured as described (47) using Fura-2/AM (Sigma–Aldrich, catalog no. F0888; 10 μm) at 340- and 380/510-nm excitation/emission wavelengths, respectively. Free Ca^2+^ concentrations were estimated by established protocols (48).

### Respirometry

Mitochondrial oxygen consumption was measured using high-resolution respirometry (O2k, Oroboros Instruments, Innsbruck, Austria) (49) as described (19).

Isolated mitochondria (0.06 mg/ml brain, 0.04 mg/ml heart) were studied in EGTA-containing or EGTA-free buffers at 30 °C. EGTA buffer (pH 7.4) contained MgCl_2_ (Sigma–Aldrich, catalog no. M2670; 5 mm), mannitol (Sigma–Aldrich, catalog no. M4125; 120 mm), MOPS (Sigma–Aldrich, catalog no. M1254; 20 mm), KH_2_PO_4_ (Sigma–Aldrich, catalog no. P5655; 5 mm), KCl (Sigma–Aldrich, catalog no. P9333; 60 mm), and EGTA (Sigma–Aldrich, catalog no. E4378; 1 mm) and was used for experiments shown in Figs. 1–5, Fig. S3 (*A* and *C*), and Fig. S5. The free Ca^2+^ concentration before stepwise increase was ∼12 nm. If not stated otherwise, substrate combinations used for experiments shown in Figs. 1–3 were malate (Sigma–Aldrich, catalog no. M1000; 2 mm) and either glutamate (Sigma–Aldrich, catalog no. 49621; 10 mm) or pyruvate (Sigma–Aldrich, catalog no. P5280; 10 mm). RR (Sigma–Aldrich, catalog no. R2751; 200 nm) was added as indicated, and state 3 was adjusted by ADP (Sigma–Aldrich, catalog no. A2754; 2 mm). Experiments shown in Fig. S3 were performed using multiple substrate inhibitor protocols as described previously (50). Substrates and inhibitors were as follows: malate (2 mm), glutamate (10 mm), ADP (2 mm), CaCl_2_ (Sigma–Aldrich, catalog no. 793639), pyruvate (10 mm), rotenone (Sigma–Aldrich, catalog no. R8875; 1.25 nm), glycerol 3-phosphate (Sigma–Aldrich, catalog no. G7886; 10 mm), succinate (Sigma–Aldrich, catalog no. W327700; 10 mm), carboxyatractyloside (Sigma–Aldrich, catalog no. C4992; 2.5 μm), antimycin A (Sigma–Aldrich, catalog no. A8674; 1 μm), ascorbate (Sigma–Aldrich, catalog no. A4034; 4 mm), TMPD (Sigma–Aldrich, catalog no. T7394; 1 mm), and azide (Sigma–Aldrich, catalog no. S8032; 5 mm).

Experiments using EGTA-free media had free Ca^2+^ concentrations of ∼600 nm before any addition of Ca^2+^ (Fig. S1 and Fig. S3 (*B, D*)) (50). Substrates and inhibitors were as follows: ADP (2 mm), glutamate (10 mm), pyruvate (10 mm), succinate (10 mm), carboxyatractyloside (2.5 μm), ascorbate (4 mm), TMPD (1 mm), and azide (5 mm). Respiratory rates measured after the addition of succinate (state 3) and after carboxyatractyloside (state 4) were used to calculate the respiratory control index.

Respiration of synaptosomes (0.4 mg of protein/ml) and thymocytes (10 × 10^6^ cells/ml) was studied in Krebs–Ringer bicarbonate buffer (KRB) at 37 °C. KRB (pH 7.4) contained NaCl (Sigma–Aldrich, catalog no. S7653; 122 mm), KCl (3.1 mm), KH_2_PO_4_ (Sigma–Aldrich, catalog no. P5655; 0.4 mm), NaHCO_3_ (Sigma–Aldrich, catalog no. S5761; 5 mm), NaTES (Sigma–Aldrich, catalog no. T0772; 20 mm), MgSO_4_ (1.2 mm), BSA (Sigma–Aldrich, catalog no. A2153; 1.6 μm), FCCP (Sigma-Aldrich, catalog no. C2920; 40 nm), glucose (Sigma, catalog no. D9434; 10 mm), lactate (Sigma, catalog no. L7022; 10 mm), or pyruvate (10 mm). Substrates and inhibitors were as follows: AOA (Sigma–Aldrich, catalog no. C13408; 2 mm), BAPTA-AM (Sigma–Aldrich, catalog no. A1076; 50 μm), cinnamate (gift of Dr. Andrew Halestrap; 250 μm), glucose (10 mm), glutamate (10 mm), lactate (10 mm), malate (2 mm), pyruvate (10 mm), and RR (200 nm).

Respiration of intact adherent fibroblasts was monitored using a Seahorse XFp Analyzer (Seahorse Bioscience). Fibroblasts were fed with either glucose (20 mm) or pyruvate (10 mm) in the presence of 100 nm FCCP and supplemented with pyruvate (10 mm) after the addition of BAPTA-AM (50 μm). All Seahorse assays were analyzed using XF Wave 2.3.0 software, according to the manufacturer's instructions. All oxygen consumption rate measurements were normalized to the protein content quantified using the Bicinchoninic Acid Protein Assay Kit (Millipore, catalog no. 71285-M) with BSA as standard.

### Measurement of mitochondrial membrane potential

Alterations in energization of isolated brain and heart mitochondria were monitored using a Cary Eclipse fluorescence spectrometer (Agilent) and the membrane potential-sensitive probe safranin O (Sigma–Aldrich, catalog no. S2255; 2 μm) as described (19). Mitochondria were incubated in EGTA media under conditions as described for respirometry. Fluorescence signals measured after uncoupling using FCCP (Sigma–Aldrich, catalog no. C2920; 1 μm) were taken as zero values, and the difference to the actual signal was calculated as a reciprocal measurement for the mitochondrial membrane potential.

### Pyruvate measurements

Pyruvate concentrations in synaptosomal incubations (2 mg of protein/ml) were measured in the presence and absence of 50 μm BAPTA-AM. 25 min after BAPTA-AM addition, the reaction was stopped by the addition of 0.4 m (final concentration) perchloric acid (Sigma–Aldrich, catalog no. 311421). Samples were centrifuged, and the supernatants were neutralized to pH 7.4 using Tris (Sigma–Aldrich, catalog no. T3253) and KOH (Merck, catalog no. 105033). Samples were used immediately for fluorimetric pyruvate determination as described elsewhere (32).

### MAS assay

Mitochondria (0.06 or 0.04 mg of protein/ml from brain or heart, respectively) were incubated in EGTA media additionally containing NADH (Sigma–Aldrich, catalog no. N1161; 250 μm), lactate (10 mm), and malate (2 mm) as described (19). Extramitochondrial Ca^2+^ was adjusted to ∼12 nm or 800 nm. The assay started with the addition of ADP (2 mm). Different substrates and inhibitors were sequentially added: (i) glutamate (2 mm), aspartate (Sigma–Aldrich, catalog no. A6558; 2 mm), and 2-oxoglutarate (Sigma–Aldrich, catalog no. 75892; 2 mm); (ii) LDH (Roth, catalog no. 6060.1; lyophilized, 5 IU/ml), MDH (Sigma, catalog no. M2634; in glycerol, 5 IU/ml), and GOT (LEE BioSolutions, catalog no. 300-20; lyophilized, 5 IU/ml); (iii) Cin (gift of Dr. Andrew Halestrap; 1 μm); and (iv) aminooxyacetate (2 mm). The assay was terminated by adding carboxyatractyloside.

### Isolation of synaptosomes

Synaptosomes from mouse brain were isolated using Percoll (Sigma–Aldrich, catalog no. P1644) gradient centrifugation as described (51, 52). The isolation buffer (pH 7.4) contained mannitol (225 mm), MOPS (20 mm), sucrose (Sigma–Aldrich, catalog no. S7903; 75 mm), EGTA (1 mm), and DTT (Sigma–Aldrich, catalog no. 43819; 0.5 mm). Final synaptosome preparations were resuspended in isolation media supplemented with 0.1 mm EGTA.

### Isolation of thymocytes

For isolation of thymocytes, thymus glands were removed and immediately put in ice-cold KRB. Thymocytes were filtered through a nylon sieve, collected, and pelleted at 1000 × *g* for 5 min. Cells were washed twice, resuspended in KRB, and kept on ice until use. Cell viability was tested by a trypan blue exclusion assay.

### Cultivation of fibroblasts

Skin fibroblasts were cultivated as described (53). Briefly, high-glucose Dulbecco's modified Eagle's media (Gibco, Life Technologies, Inc., catalog no. 41966) was supplemented with fetal bovine serum (Gibco, Life Technologies, catalog no. 10500-064; 10%), l-glutamine (Gibco, Life Technologies, catalog no. 25030-024; 2 mm), penicillin-streptomycin (Roche Applied Science, catalog no. 11074440001; 100 units/ml), ciprofloxacin (Kabi, catalog no. 15LF215F1; 4 μg/ml), and tylosin (Sigma–Aldrich, catalog no. T3397; 10 μg/ml). Passages between 3 and 6 were used for experiments.

### The isolated, perfused working rat heart

The preparation has been described in detail elsewhere (54). Briefly, rats were anesthetized using thiopental (0.15 mg/100 g body weight intraperitoneally, Inresa, catalog no. PZN-4541589). After injection of heparin (500 IU; Ratiopharm, catalog no. PZN-3029843) into the inferior vena cava, hearts were rapidly removed and placed in ice-cold Krebs–Henseleit bicarbonate buffer. The aorta was freed of excess tissue and cannulated. A brief period of retrograde perfusion (<5 min) with oxygenated buffer containing glucose (5 mm) was necessary to wash out any blood from the heart and to perform left atrial cannulation. Hearts were then perfused as working hearts at 37 °C with recirculating Krebs–Henseleit buffer (200 ml). Perfusate Ca^2+^ concentration was 2.5 mm. Hearts were perfused with glucose (5 mm) or pyruvate (5 mm; Sigma–Aldrich, catalog no. P2256-5G) as substrates. The perfusate was gassed with 95% O_2_, 5% CO_2_ and recirculated. All experiments were carried out with a preload of 15 cm H_2_O and an afterload of 100 cm H_2_O. Hearts started to beat spontaneously at a rate of ∼250 beats/min. After a period of stabilization, hearts were perfused, samples were withdrawn, and measurements were performed as indicated. Aortic and coronary flow were measured every 5 min by timing the rise of the fluid meniscus in a calibrated glass tube (54). Cardiac output was calculated as the sum of aortic and coronary flow. Heart rate was measured continuously with a Hewlett–Packard transducer and recording system (Hewlett–Packard, Waltham, MA). Mean aortic pressure (cm H_2_O) was calculated as (systolic + diastolic pressure × 2)/3. Heart rate was measured as beats/min, and cardiac output was measured as ml/min. Cardiac power was determined as described elsewhere (55). The MAS was inhibited with AOA (0.1 mm; Sigma–Aldrich, catalog no. C13408-1G). Glucose was added to exclude a decreased availability as limiting factor. Pyruvate was used as malate-aspartate shuttle–independent substrate, and cinnamate (0.75 mm; Sigma–Aldrich, catalog no. C2020-10G) was used to inhibit the mitochondrial PC. At the end of perfusion, hearts were frozen and weighed. The wet to dry ratio was determined, and the dry weight was calculated.

### Substrate oxidation rates

Samples of the coronary effluent (2 ml) were withdrawn every 5 min for the assessment of glycolysis rates determined as the production of ^3^H_2_O from [2-^3^H]glucose (Perkin-Elmer Life Sciences, catalog no. NET331C) (56).

### Statistical analyses

Statistical analyses were performed using GraphPad Prism (GraphPad Software, version 8 for Mac OS X) for comparisons as indicated of at least *n* = 3 independent experiments. All data are shown as the mean. *Error bars* represent S.E. A *p* value of <0.05 was considered statistically significant.

## Author contributions

M. Szibor, W. S. K., and F. N. G. conceptualization; M. Szibor, Z. G., T. G., T. E., G. D.-V., M. K., N. K., K. H., F. S., A. B., M. Schwarzer, V. L., and F. N. G. data curation; M. Szibor, F. S., and F. N. G. visualization; M. Szibor, W. S. K., and F. N. G. writing-original draft; M. Szibor, Z. G., T. G., T. E., G. D.-V., M. K., N. K., K. H., F. S., A. B., M. Schwarzer, T. D., H.-J. H., V. L., S. V., W. S. K., and F. N. G. writing-review and editing; Z. G., T. G., T. E., G. D.-V., M. K., N. K., K. H., A. B., M. Schwarzer, V. L., W. S. K., and F. N. G. formal analysis; T. D., H.-J. H., S. V., and F. N. G. funding acquisition; F. N. G. supervision; F. N. G. validation; F. N. G. investigation; F. N. G. methodology; F. N. G. project administration.

## Supplementary Material

Supporting Information

## Footnotes

3 The abbreviations used are:

OXPHOS: oxidative phosphorylation
α-OG: α-oxoglutarate
AOA: GOT inhibitor aminooxyacetate
Cin: cinnamate
GC1 and GC2: glutamate carrier 1 and 2, respectively
GOT: glutamate oxaloacetate transaminase
KRB: Krebs–Ringer bicarbonate buffer
LDH: lactate dehydrogenase
MAS: malate-aspartate shuttle
MDH: malate dehydrogenase
RR: ruthenium red
TMPD: *N*,*N*,*N*′,*N*′-tetramethyl-*p*-phenylenediamine
PDH: pyruvate dehydrogenase
KO: knockout
MCU: mitochondrial Ca^2+^ uniporter
PC: pyruvate carrier
FCCP: carbonyl cyanide *p*-trifluoromethoxyphenylhydrazone
ANOVA: analysis of variance.
