# Supplementary material for: Cytosolic, but not matrix, calcium is essential for adjustment of mitochondrial pyruvate supply
Source: J Biol Chem. 2020 Feb 24;295(14):4383–97. doi: 10.1074/jbc.RA119.011902 (PMC7135991; doi:10.1074/jbc.RA119.011902)
Supplement: Supporting Information [file supp_295_14_4383__index.html]

Cytosolic, but not matrix, calcium is essential for adjustment of mitochondrial pyruvate supply — EDITORS' PICK: Cytosolic calcium controls mitochondrial pyruvate supply — Supporting Information 

# Cytosolic, but not matrix, calcium is essential for adjustment of mitochondrial pyruvate supply

## Supporting Information

- Supporting Information (to be published online) - Supporting Information. Supplementary Figures S1-S5.
