# Supplementary material for: Cytosolic, but not matrix, calcium is essential for adjustment of mitochondrial pyruvate supply
Source: J Biol Chem. 2020 Feb 24;295(14):4383–97. doi: 10.1074/jbc.RA119.011902 (PMC7135991; doi:10.1074/jbc.RA119.011902)
Supplement: Supporting Information [file supp_RA119.011902_157130_2_supp_478255_q5ylvy.pdf]

Cytosolic, but not matrix, calcium is essential for adjustment of mitochondrial pyruvate supply

Szibor, Gizatullina et al.

Supplementary Figures

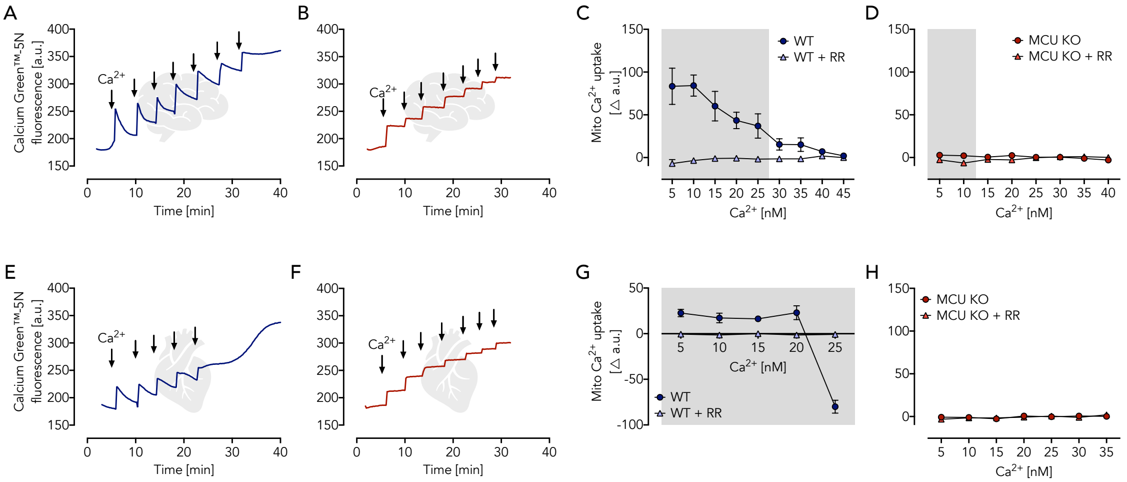

Figure S1. Measurement of Ca^{2+} uptake kinetics using brain and heart mitochondria isolated from WT and MCU KO mice. (A, B) Representative Ca^{2+} uptake traces of brain mitochondria (0.1 mg/ml) incubated in EGTA-free medium energized with glutamate (10 mM) and malate (2 mM) using Calcium Green-5N fluorescent probe. Arrows indicate Ca^{2+} additions. (C, D) Ca^{2+} uptake quantification for brain mitochondria in presence or absence of the MCU inhibitor ruthenium red (RR) as indicated plotted against total amount of added Ca^{2+}. Data are shown as mean ± SEM of n = 5 experiments. Grey areas in C and D denote range of significant difference with $p < 0.05$ between WT and WT + RR, and MCU KO and MCU KO + RR mitochondria determined using two-way ANOVA analysis with Sidak's multiple comparisons post-hoc test. (E, F) Ca^{2+} uptake traces of heart mitochondria (0.1 mg/ml) under conditions as described for A and B. (G, H) Ca^{2+} uptake quantification for heart mitochondria in presence or absence of RR as indicated. Data are shown as mean ± SEM of n = 5 experiments. Grey area in G denotes range of significant difference with $p < 0.05$ between WT and WT + RR mitochondria determined using two-way ANOVA analysis with Sidak's multiple comparisons post-hoc test.

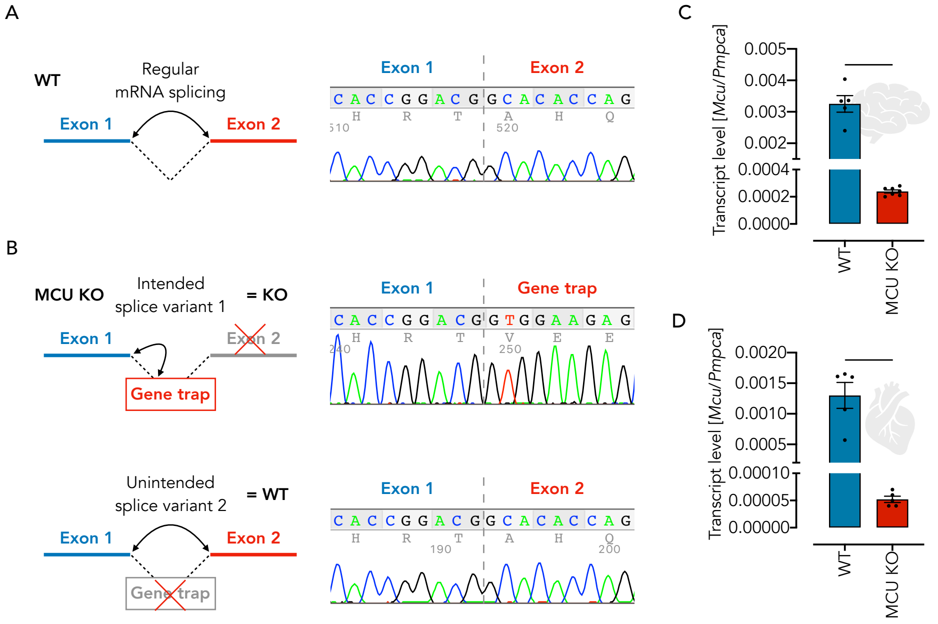

Figure S2. Quantification of *Mcu* transcripts. (A) Schematic of regular exon splicing in WT RNA encoding the *Mcu* transcript. (B) Schematic of exon splicing in MCU KO animals. Note, MCU KO animals express residual amounts of WT *Mcu* mRNA transcripts due to unintended deletion of the gene trap (unintended splice variant 2). (C) *Mcu* transcript level in hippocampus determined by quantitative PCR with *mitochondrial processing peptidase* (*Pmpca*) used as reference gene. Data are shown as mean ± SEM of WT n = 5 and MCU KO n = 7 experiments. Horizontal bar denotes a significant difference with $p < 0.05$ determined using Student's *t* test. (D) *Mcu* transcript level in heart tissue. Data are shown as mean ± SEM of n = 5 experiments. Horizontal bar denotes a significant difference with $p < 0.05$ determined using Student's *t* test.

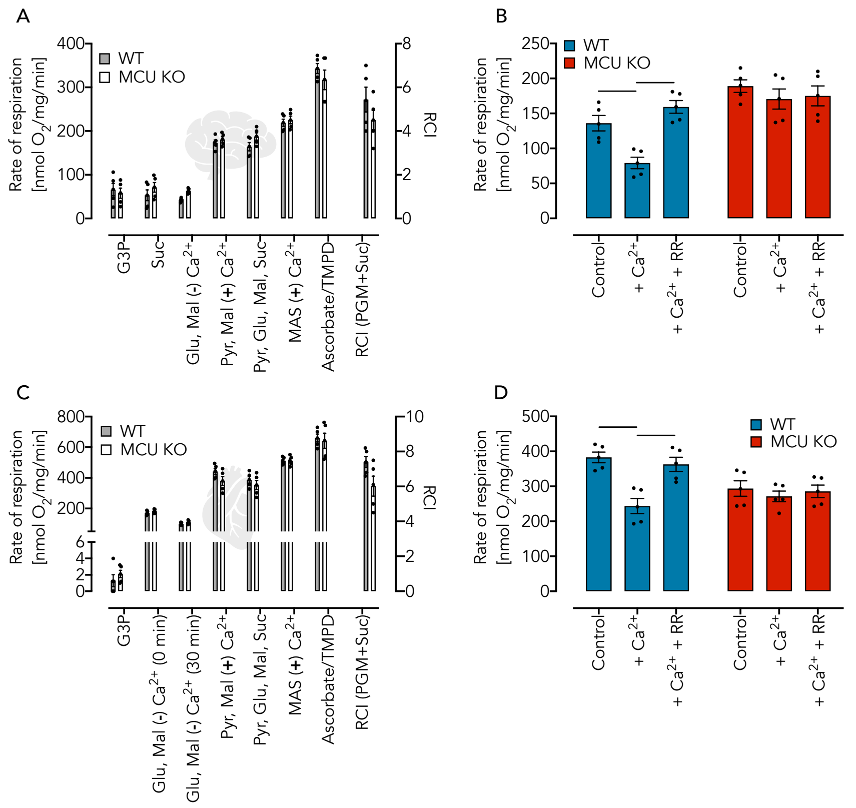

Figure S3. Measurement of substrate-specific respiratory rates and assessment of Ca^{2+} overload effects on respiratory rates using isolated WT and MCU KO brain and heart mitochondria. (A) Brain (0.06 mg/ml) and (C) heart (0.04 mg/ml) mitochondria incubated in EGTA medium in the presence of ADP (2 mM) and substrate combinations as indicated. G3P (glycero-3-phosphate); Suc (succinate plus rotenone); Glu, Mal (-) Ca^{2+} (glutamate, malate without Ca^{2+}); Pyr, Mal (+) Ca^{2+} (pyruvate, malate plus Ca^{2+} 800 nM); Pyr, Glu, Mal, Suc (pyruvate, glutamate, malate, succinate); MAS (+) Ca^{2+} (glutamate, malate, lactate, NADH, aspartate, α-oxoglutarate, LDH, MDH, GOT plus 800 nM Ca^{2+}); ascorbate/TMPD; RCI (respiratory control index calculated from respiratory rates for glutamate, malate, pyruvate, succinate plus 600 nM Ca^{2+} in state 3 and state 4); Glu, Mal (-) Ca^{2+} (0 min) (glutamate, malate without Ca^{2+} without preincubation); Glu, Mal (-) Ca^{2+} (30 min) (glutamate, malate without Ca^{2+} measured after 30 minutes preincubation). (B) Respiratory rates measured in brain and (D) heart mitochondria incubated in EGTA-free medium energized by pyruvate (10 mM), glutamate (10 mM), malate (2 mM) and succinate (10 mM) in the presence of ADP (2 mM, state 3). Addition of Ca^{2+} (20 µM) and ruthenium red (RR, 200 nM) as indicated. All data shown as mean ± SEM of n = 5 experiments. Horizontal bars denote significant differences with $p < 0.05$ for comparisons as indicated, determined using two-way ANOVA analysis with Tukey's multiple comparisons test.

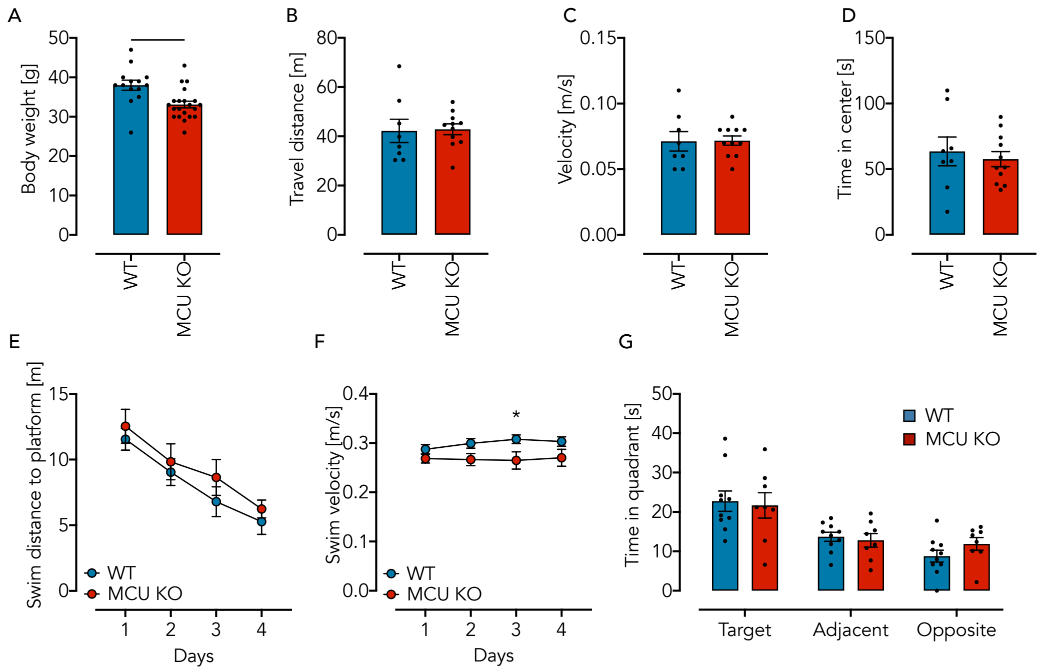

Figure S4. Assessment of cognitive abilities, anxiety levels and endurance in WT and MCU KO mice. (A) Body weight. Data are shown as mean ± SEM of WT n = 14 and MCU KO n = 21 mice. The horizontal bar indicates a significant difference with $p < 0.05$ analyzed using Student's *t*-test. (B-D) Open Field tests. Data are shown as mean ± SEM of WT n = 8 and MCU KO n = 11 mice. (B) Traveling distance. (C) Velocity/average speed. (D) Time intervals spent the center region of the open field arena (anxiety test). (E-G) Morris Water Maze tests. Data are shown as mean ± SEM of WT n = 10 and MCU KO n = 8 mice. (E) Swimming distance (until reaching a hidden platform). (F) Swimming velocity. *$p < 0.05$ determined using two-way ANOVA analysis with Sidak's multiple comparisons post-hoc test. (G) Time spent in target quadrant during the probe trial (no hidden platform).

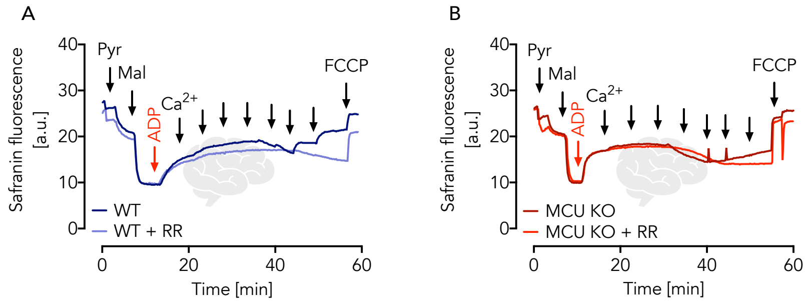

Figure S5. Assessment of pyruvate-dependent mitochondrial membrane potential in brain mitochondria isolated from WT and MCU KO mice. Representative membrane potential traces from (A) WT and (B) MCU KO mitochondria incubated in EGTA-medium using safranin O (2 µM) as fluorescent probe. Additions of ruthenium red (RR, 200 nM), pyruvate (Pyr, 10 mM), malate (Mal, 2 mM), ADP (2 mM), Ca^{2+} (arrows indicate step-wise increase from approximately 12 nM to 2 µM) and FCCP (1 µM) as indicated. Note, Ca^{2+} overload induces toxicity and loss of membrane potential in WT but not in MCU KO or RR-treated WT mitochondria.
